# Chinese herb feed additives improved the growth performance, meat quality, and nutrient digestibility parameters of pigs

**DOI:** 10.1002/ame2.12104

**Published:** 2020-03-16

**Authors:** Zhong‐ning Lin, Li Ye, Zhen‐wu Li, Xiu‐sheng Huang, Zheng Lu, You‐quan Yang, Huan‐wei Xing, Jie‐ying Bai, Zhao‐yang Ying

**Affiliations:** ^1^ Agricultural Ecology Institute Fujian Academy of Agricultural Sciences Fujian Engineering and Technology Research Center for Hilly Prataculture Fuzhou P.R. China; ^2^ State Key Laboratory of Pathogen and Biosecurity Beijing Institute of Microbiology and Epidemiology Beijing Key Laboratory of Vector Borne and Natural Focus Infectious Diseases Beijing P.R. China; ^3^ The Institute of Molecular Medicine Peking University Beijing P.R. China; ^4^ Institute of Biophysics School of Sciences Hebei University of Technology Tianjin P.R. China

**Keywords:** Chinese herb feed additive, growth performance, meat quality, pig

## Abstract

**Background:**

Since the use of antibiotics in animal feed has become a critical concern worldwide due to severe threats to human health and environment, we are in need of finding alternatives to antibiotics in pig breeding, maintaining the health of pigs, and getting high‐quality pork. As traditional Chinese herbs (TCH) are rich natural resources in China and show great benefits to human health we propose to transfer this abundant resource into animal production industry as additives.

**Methods:**

Three groups of Chinese herbs (groups A, B, and C) were used as feed additives in the diet for pigs. In total 32 pigs were arranged in four groups (groups A, B, C, and control group, NC), fed in the same facility, eight pigs (one group) in each colony, free drinking, for 120 days. The feed:gain ratio (F/G), meat quality, total protein, and amino acid concentration of muscle were checked in the experiments.

**Results:**

After 120 days of feeding, the feed:gain ratio (F/G) of pigs in groups A, B, and C was decreased 17.56%, 9.31%, and 13.86% compared with NC treatment, respectively. The diets supplemented with Chinese herbs improved meat quality, increased loin eye area (especially group A and C showed significant difference, *P* < .001), the total protein (increased ratio vs NC was A = 4.54%, B = 0.38% and C = 3.53%), amino acid concentration of muscle, increased the villus height:crypt depth ratio, and induced positive effects on serum biochemical parameters and immune function (serum TC and TG concentrations were significantly lower than those in the NC group, *P* < .05.).

**Conclusions:**

The use of Chinese herbal feed additives can reduce the cost of pig breeding and produce high‐quality pock. The combination of these effects would contribute to better absorption ability of the intestinal tract and yield a better growth performance.

## 1 INTRODUCTION

Antibiotics have long been of great benefit for people, both in the medical treatment of human disease and in animal food, where they improve the growth performance and feed utilization during animal production.^1^ Antibiotics as in‐feed supplements affect all stages of pork production, including the gestation, nursing, growing, and terminal stage, although the effects show stage‐dependent differences.^1^ However, the use of antibiotics in animal feed has become a critical concern worldwide due to the severe threats to human health and the environment.^1^, ^2^, ^3^ Therefore, many researchers have investigated alternative feed additives or supplements, such as probiotics, enzymes, minerals, organic acids, and herbs.^3^ Phytogenic comprise a wide variety of herbs, spices, and their derived products, especially essential oils.^4^, ^5^ The available evidence indicates that phytogenic compounds may specifically enhance the activities of digestive enzymes and nutrient absorption.^4^ In addition, some phytogenic compounds seem to promote intestinal mucus production.^4^

Traditional Chinese herbs (TCH) have been used for more than 3000 years.^6^ As TCH are rich natural resources in China and show great benefits to human health we propose to transfer this abundant resource into animal production industry as additives. Recently, Chinese herbs are garnering growing interest as feed additives in animal production, but data on the effects of Chinese herbs on swine production are scarce.^7^ In thi study, three groups of Chinese herbs were used as feed additives in the diet of swine. The herbs were put in the feed for 120 days, and growth performance, meat quality, serum biochemical parameters, intestinal villus morphology, and gut microbiota were investigated. The results confirm the feasibility of Chinese herbs as feed additives in the production of pigs.

## 2 MATERIALS AND METHODS

### 2.1 Preparation of Chinese herb feed additive

A total of three combinations of Chinese herb feed additives were designed: group A (semen raphani, atractylodes, coix seed, poria, crataegus, codonopsis pilosula); group B (sophora japonica, pulsatilla chinensis regel, portulacae, atractylodes, semen euryales, radix scutellariae); and group C (copperleaf herb, astragalus, atractylodes, coptidis rhizoma, pseudollaria heterophylla, dried orange peel).

### 2.2 Experimental design, animals, housing, and diets

The animal protocol for this experiment was approved by the Animal Care Committee of Fujian Academy of Agricultural Sciences, China. Animals were maintained and processed in accordance with the Fujian Academy of Agricultural Sciences Guide for the Care and Use of Laboratory Animals.

Antibiotic‐free basal diet was purchased from Hualong Group, Fujian province, China (Table 1). A total of 32 swine with an average initial body weight (BW) of 38 kg were used in a 120‐day experiment. The swine were housed in an environmentally controlled, slatted‐floor facility in adjacent pens (1.2‐1.5 m^2^) and were allowed ad libitum access to feed and water through a self‐feeder and nipple drinker throughout the experimental period. Swine were randomly allocated into four groups (eight pigs per group, including four males and four females). Piglets in the control group were fed a basal diet that was formulated based on a corn powder and soybean meal diet that met the National Research Council (2012) swine requirements and China swine standards. Dietary treatments included negative control (NC, antibiotic‐free basal diet), group A (NC + Chinese herb feed additive group A), group B (NC + Chinese herb feed additive group B), and group C (NC + Chinese herb feed additive group C). Each group of Chinese herb feed additives was smashed, mixed, and directly added into the basal diet.

**Table 1:** Ingredient composition and analyzed nutrient contents of antibiotic‐free basal diet

	30‐60 (kg)	60‐120 (kg)
Corn (%)	65	65
Wheat bran (%)	10	13
Soybean meal (%)	20	17
Premix compound (%)	5	5
Total (%)	100	100
Digestible energy (MJ/kg)	12.97	12.97
Crude protein (%)	16.4	14.5
Lysine (%)	0.82	0.7
Calcium (%)	0.55	0.7
Phosphorus (%)	0.45	0.4

### 2.3 Sampling and measurements

Piglets were weighed at the beginning and end of this dietary experiment to obtain information about average daily gain (ADG). Feed added to the feeder and any wasted feed were weighed to calculate average daily feed intake (ADFI). The feed:gain ratio (F/G) was further calculated based on the ADG and ADFI data.

To measure serum biochemical parameters, blood samples were collected via jugular venipuncture into vacuum tubes containing no additive at the end of the experiment. The serum was separated by centrifugation for 30 minutes at 2000 g at 4°C, and the aliquot was stored at 4°C (within 24 hours) prior to assay. The levels of serum total protein (TP), globulin (GLO), albumin (ALB), blood urea nitrogen (BUN), alkaline phosphatase (ALP), glucose (GLU), triglycerides (TG), total cholesterol (TC), glutamic‐pyruvic transaminase (ALT), glutamic oxalacetic transaminase (AST), glutamyltranspeptidase (GTP), serum creatinine (CREA), high‐density lipoprotein (HDLC), low‐density lipoprotein (LDLC), and serum amylase (AMS) were analyzed by an AU480 Chemistry System (Beckman).

Pigs were stunned using electronarcosis and killed at the end of the experiment. Meat quality and amino acid levels in meat were measured. The duodenum, jejunum, ileum, and intestines were sampled and examined for morphology as well as intestinal microflora.

### 2.4 Statistical analysis

For each set of assays, at least three independent experiments were carried out. The results are expressed as the means ± standard deviations (SD). Statistical significance was calculated using the t test, followed by comparing the additive‐treated groups with the NC group. Statistical analysis was carried out using GraphPad Prism software version 5 (GraphPad Software). The results were statistically significant at *P* < .05.

## 3 RESULTS

### 3.1 Growth performance

The effects of Chinese herb feed additives on the growth performance of pigs are shown in in Table 2. Pigs in the Chinese herb feed additive groups showed greater ADG and lower F/G compared with NC treatment. The F/G of pigs in groups A, B, and C was decreased 17.56%, 9.31%, and 13.86% compared with NC treatment. The ADG of pigs in group A was higher than that in groups B and C, while the F/G of pigs in group A was lower than groups B and C. There was no difference in ADFI between herb‐treated groups and the control group. These results indicate that three Chinese herb feed additive mixtures can enhance the growth performance of swine.

**Table 2:** Effects of different Chinese herb feed additive treatments on the growth performance of swine

	Group A	Group B	Group C	NC
Total feed (kg)	371.28	363.48	372.36	366.36
ADFI (kg/pig)	3.09	3.03	3.10	3.05
Total Chinese herb feed additive (kg)	3.65	3.65	3.65	0
Ratio of Chinese herb feed additive (%)	0.98	1.00	0.98	0
Initial BW (kg)	38.52 ± 5.38	38.20 ± 2.84	37.78 ± 3.55	37.92 ± 3.18
Final BW (kg)	137.75 ± 7.60*	126.75 ± 8.59	133.00 ± 6.18**	118.75 ± 7.26
Average gain (kg)	99.22 ± 3.81***	88.55 ± 6.29*	95.22 ± 2.89***	80.82 ± 4.46
ADG (kg/pig)	0.83 ± 0.03***	0.74 ± 0.05*	0.79 ± 0.02***	0.67 ± 0.04
F/G	3.75 ± 0.15**	4.12 ± 0.31*	3.91 ± 0.12***	4.54 ± 0.26
F/G (compared with NC)	↓17.56% (0.79)	↓9.31% (0.42)	↓13.86% (0.63)	

Abbreviations: ADFI, average daily feed intake; ADG, average daily gain; BW, body weight; NC, negative control; F/G, feed/gain ratio.

* *P* < .05.

** *P* < .01.

*** *P* < .001.

### 3.2 Meat quality and amino acid levels

The qualitative characteristics of the meat quality are shown in Table 3. The loin eye area tended to be increased in pigs fed with the Chinese herb feed additives. Specifically, the loin eye area was significantly increased in groups A and C compared with the control group. In contrast, the drip loss rate tended to be decreased in the Chinese herb feed additive diet groups, and the drip loss rate of groups A and C was significantly decreased compared with the control group. The pH1, pH24, and cooked meat percentage did not differ significantly between the groups (Table 3). Total protein, total amino acids, total lipids, crude ash, calcium, and phosphorus in the meat of pigs treated with Chinese herb feed additives tended to be increased over the control group. The total protein of pigs in groups A, B, and C was increased by 4.54%, 0.38%, and 3.53%, respectively. The total amino acids of pigs in groups A, B, and C were increased by 5.67%, 1.44%, and 3.94%, respectively. The total lipid of pigs in groups A, B and C was increased by 14.19%, 12.84%, and 17.57%, respectively. The crude ash of pigs in groups A, B, and C was increased by 1.38%, 8.00%, and 9.80%, respectively. The calcium of pigs in groups B and C was increased by 3.70% and 1.01%, respectively, while it was decreased in group A by 4.04%. The phosphorus of pigs in groups B and C was increased by 1.47% and 4.12%, respectively, while it was significantly decreased in group A by 7.47% (*P* < .001). These results indicate that dietary feed supplemented with Chinese herbs can improve the meat quality of swine.

**Table 3:** Effects of different Chinese herb feed additive treatments on the meat quality of swine

	Group A	Group B	Group C	NC
Loin eye area (cm^2^)	156.10 ± 8.48^a^	152.80 ± 11.58^ab^	159.50 ± 15.61^a^	138.20 ± 7.43^b^
PH1	6.38 ± 0.19	6.34 ± 0.29	6.13 ± 0.22	6.09 ± 0.19
PH24	5.51 ± 0.14	5.47 ± 0.17	5.52 ± 0.28	5.76 ± 0.17
Drip loss rate (%)	2.27 ± 0.25^a^	2.67 ± 0.76^ab^	2.14 ± 0.29^a^	3.24 ± 0.84^b^
Cooked meat percentage (%)	27.96 ± 1.08	27.53 ± 1.22	27.71 ± 1.39	26.81 ± 1.01
Total protein (g/100 g)	82.85 ± 1.05^a^	79.55 ± 2.05^b^	82.05 ± 1.85^ab^	79.25 ± 0.65^b^
Total amino acids (g/100 g)	77.32 ± 0.98	74.23 ± 1.88	76.06 ± 2.02	73.175 ± 1.32
Total lipid (g/100 g)	8.45 ± 0.35	8.35 ± 1.15	8.70 ± 1.90	7.40 ± 1.50
Crude ash (g/100 g)	5.90 ± 0.261	6.28 ± 0.24	6.38 ± 0.44	5.82 ± 0.15
Calcium (mg/100 g)	1.42 ± 0.29	1.54 ± 0.13	1.50 ± 0.13	1.48 ± 0.02
Phosphorus (mg/100 g)	671.28 ± 9.14^b^	736.15 ± 6.394^a^	755.36 ± 10.20^a^	725.50 ± 23.84^a^

Note

Different letters (a, b) indicate differences between groups (*P* < .05).

The amino acid concentration in muscle is shown in Table 4. The concentration of total amino acids showed a significant increase in the Chinese herb feed additive treatment groups compared with the control group, with 5.67%, 1.44%, and 3.94% increases, respectively. The concentration of essential amino acids increased 6.75%, 1.38%, and 4.99% in the treatment groups compared with the control group, respectively. The concentration of isoleucine showed a significant increase in the treatment groups compared with the control group. The concentrations of valine, phenylalanine, aspartic acid, glutamic acid, glycine, alanine, and arginine in muscle of group A were significantly increased in comparison to control swine. The concentrations of methionine, leucine, and proline in muscle of groups B and C were significantly increased in comparison to control pigs. The ratios of EAA/TAA and EAA/NEAA were 0.4 and 0.65, which were higher than the FAO/WHO standard. The concentration of aromatic amino acids increased 7.47%, 1.87%, and 4.20% in groups A, B, and C, respectively. The concentration of delicious amino acids increased 4.40%, 1.32% and 2.54%, respectively, in the Chinese herb feed additive groups. These results indicate that Chinese herb feed additives can enhance amino acid concentrations in the muscle of swine. Group A was the best among the three investigated Chinese herb feed additives in improving meat quality.

**Table 4:** Effects of different Chinese herb feed additive treatments on meat amino acid levels of swine (g/100 g)

	Group A	Group B	Group C	NC
Thr	3.78 ± 0.04	3.70 ± 0.08	3.68 ± 0.16	3.65 ± 0.03
Val	4.02 ± 0.04***	3.89 ± 0.13	3.96 ± 0.16	3.84 ± 0.01
Met	1.95 ± 0.14**	1.42 ± 0.06	1.96 ± 0.07*	1.37 ± 0.34
Ile	3.70 ± 0.05**	3.60 ± 0.11**	3.70 ± 0.12**	3.49 ± 0.09
Leu	6.33 ± 0.04**	6.10 ± 0.14	6.21 ± 0.17*	5.98 ± 0.07
Lys	6.96 ± 0.06	6.72 ± 0.19	6.92 ± 0.31	6.70 ± 0.11
Phe	4.25 ± 0.03**	4.00 ± 0.08	4.06 ± 0.25	4.00 ± 0.08
Tyr	2.66 ± 0.01	2.54 ± 0.11	2.54 ± 0.01	2.42 ± 0.11
Cys	0.61 ± 0.05	0.62 ± 0.08	0.65 ± 0.04	0.62 ± 0.01
Asp	7.56 ± 0.12***	7.34 ± 0.20	7.40 ± 0.19	7.27 ± 0.10
Glu	12.56 ± 0.10**	12.20 ± 0.38	12.20 ± 0.29	12.02 ± 0.03
Gly	3.48 ± 0.06***	3.34 ± 0.10	3.53 ± 0.10	3.30 ± 0.05
Ala	4.52 ± 0.04**	4.40 ± 0.08	4.45 ± 0.18	4.32 ± 0.04
Arg	5.10 ± 0.05***	4.96 ± 0.14	5.06 ± 0.08	4.92 ± 0.052
His	3.67 ± 0.06	3.52 ± 0.16**	3.60 ± 0.32	3.48 ± 0.17
Ser	3.09 ± 0.07	2.96 ± 0.06	2.98 ± 0.06	2.94 ± 0.04
Pro	3.08 ± 0.05*	2.90 ± 0.09	3.07 ± 0.04**	2.84 ± 0.03
Total amino acids (TAA)	77.32 ± 0.98**	74.23 ± 1.88*	76.06 ± 2.02*	73.18 ± 1.328
TAA compared with NC	+5.67%	+1.44%	+3.94%	
Essential amino acids (EAA)	30.10 ± 0.39	29.44 ± 0.674	30.48 ± 1.15	29.04 ± 0.76
EAA compared with NC	+6.75%	+1.38%	+4.99%	
EAA/TAA	0.4009	0.3965	0.4008	0.3968
EAA/NEAA	0.6691	0.6571	0.6689	0.6578
Aromatic amino acid (AAA)	6.90 ± 0.35	6.54 ± 0.35	6.70 ± 0.26	6.42 ± 0.19
AAA compared with NC	+7.47%	+1.87%	+4.20%	
Delicious amino acid (DAA)	33.22 ± 0.36	32.24 ± 0.90	32.64 ± 0.42	31.82 ± 0.18
DAA compared with NC	+4.40%	+1.32%	+2.54%	

* *P* < .05.

** *P* < .01.

*** *P* < .001.

### 3.3 Serum biochemical parameters

The results of serum biochemical parameters are given in Table 5. Serum TP, ALB, and A/G were increased in the Chinese herb feed additive groups compared with the control group, while GLO was decreased in the treated groups. These results indicate that the Chinese herb feed additives showed beneficial effects on immune function and liver synthetic function in pig. Furthermore, serum ALT and AST levels in groups A, B, and C were significantly decreased compared with the NC group. Serum ALP and GLU levels were higher and GTP lower compared with the NC group. These results indicate that the Chinese herb feed additives can relieve damage to the liver, gallbladder, skeletal muscle, and heart muscle. Serum TC and TG concentrations in the treated groups were significantly lower than those in the NC groups (*P* < .05). Moreover, serum AMS and LDLC were lower than in the control group. Finally, serum BUN levels were decreased, while CREA was increased, compared with the control group. Together, these results suggest that diets supplemented with Chinese herb feed additives could induce positive effects on serum biochemical parameters in swine.

**Table 5:** Effects of different Chinese herb feed additive treatments on serum biochemical parameters in swine

	Group A	Group B	Group C	NC
TP (g/L)	70.70 ± 2.60	66.20 ± 4.80	70.40 ± 5.60	66.60 ± 3.00
ALB (g/L)	52.40 ± 2.50	51.60 ± 3.70	48.70 ± 0.80*	50.00 ± 1.00
GLO (g/L)	17.00 ± 1.90	15.20 ± 5.50	19.70 ± 3.80	16.40 ± 2.90
A/G	3.10 ± 0.40	3.80 ± 1.40*	2.60 ± 0.50	3.10 ± 0.50
ALT (U/L)	49.50 ± 10.50	50.20 ± 16.90	52.00 ± 4.70	56.00 ± 9.80
AST (U/L)	38.80 ± 21.20	39.00 ± 13.00*	33.80 ± 10.30**	60.00 ± 25.00
AST/ALT	0.80 ± 0.20	0.80 ± 0.40	0.70 ± 0.20**	1.10 ± 0.60
ALP (U/L)	126.50 ± 36.60	124.80 ± 30.20	126.80 ± 37.30	118.50 ± 25.30
GTP (U/L)	32.50 ± 20.30	34.00 ± 9.80	36.80 ± 14.90	45.50 ± 10.80
BUN (mM)	3.48 ± 0.41	3.32 ± 0.42	4.36 ± 0.96	3.43 ± 0.41
CREA (µM)	128.30 ± 5.60	134.40 ± 12.00	121.40 ± 11.10	123.00 ± 7.60
TC (mM)	2.15 ± 0.37*	2.10 ± 0.14***	1.90 ± 0.36**	2.90 ± 0.28
TG (mM)	0.48 ± 0.05*	0.42 ± 0.05*	0.42 ± 0.13	0.55 ± 0.06
HDLC (mM)	1.10 ± 0.14	1.02 ± 0.08	1.03 ± 0.05	1.05 ± 0.07
LDLC (mM)	1.08 ± 0.07	1.07 ± 0.15	1.10 ± 0.12	1.24 ± 0.23
GLU (mM)	4.65 ± 0.44*	4.47 ± 0.20**	4.22 ± 0.75	4.09 ± 0.14
AMS (U/L)	2127.80 ± 110.40**	2676.80 ± 916.00	2706.80 ± 916.00	3062.20 ± 512.90
K (mM)	4.65 ± 0.27	5.11 ± 0.49	4.90 ± 0.48	4.70 ± 0.59
Na (mM)	145.50 ± 1.20	146.80 ± 0.80**	146.80 ± 1.30*	145.30 ± 1.00

Abbreviations: A/G, albumin/globulin ratio; ALB, albumin; ALP, alkaline phosphatase; ALT, glutamic‐pyruvic transaminase; AMS, serum amylase; AST, glutamic‐oxalacetic transaminase; BUN, blood urea nitrogen; CREA, creatinine; GTP, glutamyltranspeptidase; GLO, globulin; GLU, glucose; HDLC, high‐density lipoprotein; LDLC, low‐density lipoprotein; TC, total cholesterol; TG, triglycerides; TP, total protein.

* *P* < .05.

** *P* < .01.

*** *P* < .001.

### 3.4 Intestinal villi morphology

**FIGURE 1:**
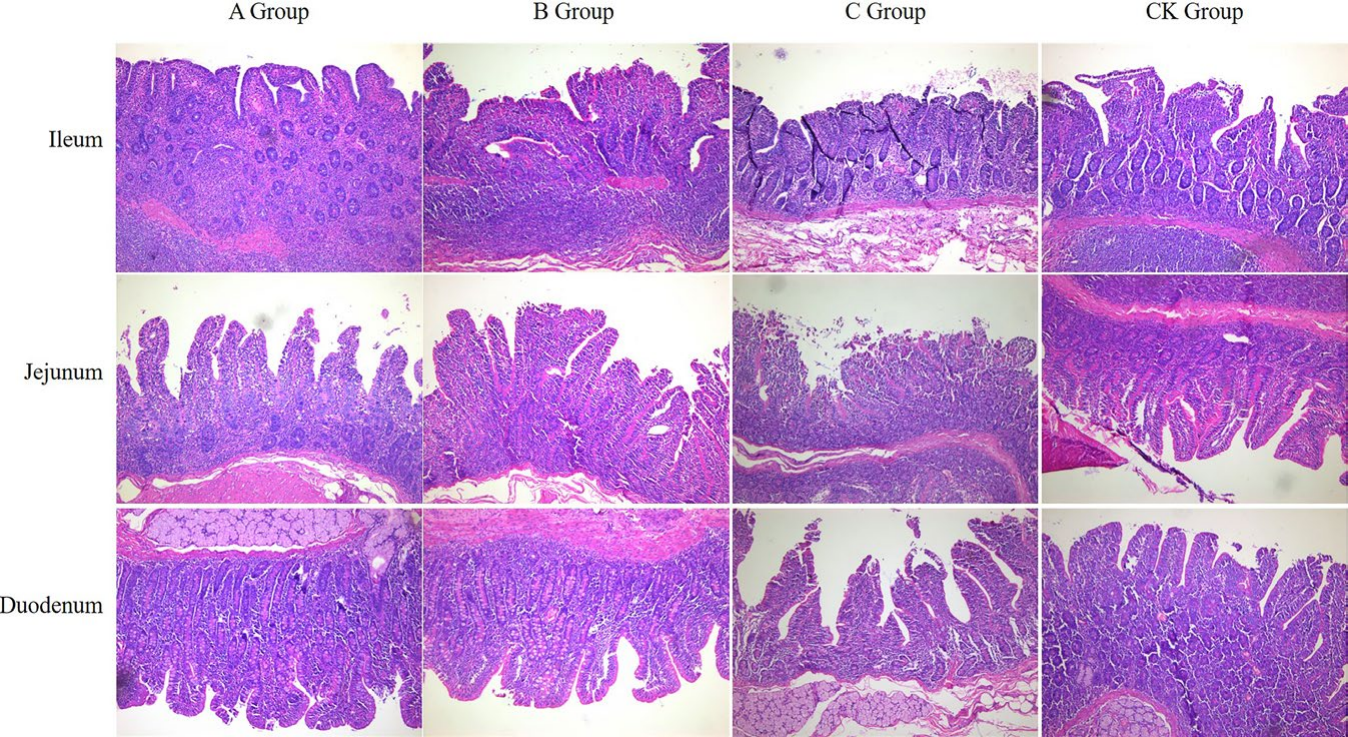
Immunohistochemistry of the ileum, jejunum, and duodenum of swine. The intestinal tissues were dehydrated to make paraffin slides, which were stained with hematoxylin‐eosin (HE). Under an optical microscope (100×), images of the ileum, jejunum, and duodenum were captured. Scale bar: 500 µm

Dietary supplementation with Chinese herb feed additive increased the villus height:crypt depth ratio in the ileum, jejunum, and duodenum compared to the NC (Table 6 and Figure 1). Dietary Chinese herb feed additive supplementation tended to reduce crypt depth in the duodenum and jejunum. There was no effect on villus height in the intestinal segments of any treatment group. These results indicate that Chinese herb feed additive can improve the nutrient digestion and absorption capacity of the small intestine.

**Table 6:** Effects of different Chinese herb feed additive treatments on small intestinal morphology in swine

	Group A	Group B	Group C	Group NC
Ileum				
Villus height, (µm)	362.93 ± 53.38	333.34 ± 35.12	336.39 ± 32.52	324.11 ± 63.80
Crypt depth, (µm)	336.26 ± 19.25	338.05 ± 37.49	328.50 ± 36.91	370.10 ± 73.46
Villus height:crypt depth, (μm:μm)	1.08 ± 0.12	1.00 ± 0.21	1.05 ± 0.16	0.90 ± 0.26
Jejunum				
Villus height, (µm)	278.74 ± 52.16	272.54 ± 50.46	278.13 ± 43.66	256.89 ± 84.36
Crypt depth, (µm)	257.44 ± 50.86	290.20 ± 99.08	249.75 ± 58.97	287.27 ± 85.78
Villus height:crypt depth, (μm:μm)	1.14 ± 0.25	1.03 ± 0.35	1.14 ± 0.16	0.90 ± 0.17
Duodenum				
Villus height, (µm)	309.49 ± 59.08	286.28 ± 58.05	301.14 ± 68.55	273.92 ± 57.19
Crypt depth, (µm)	276.42 ± 42.20	264.45 ± 43.56	264.61 ± 54.32	280.07 ± 68.74
Villus height:crypt depth, (μm:μm)	1.15 ± 0.32	1.08 ± 0.13	1.14 ± 0.28	0.99 ± 0.07

## 4 DISCUSSION

Phytogenic feed additives, referring to essential oils, spices, herbs or plant extracts, combine bioactive ingredients and flavoring substances, which have recently been gaining considerable interest due to their ability to improve performance by sustaining a healthy gut environment.^5^ Furthermore, the phytogenic feed additives can improve the flavor and palatability of feed and increase the activity of digestive enzymes of the gastrointestinal tract and nutrient utilization in pigs.^8^ Among phytogenic feed additives, Chinese herbs are the most economical and labor‐effective additive due to their simple preparation and low cost. Fermented herb additives, such as water plantain, red ginseng, green tea, and *Ginkgo biloba *L. (GBL), can improve the growth performance, feed efficiency, and nutrient digestibility in pigs.^8^, ^9^, ^10^, ^11^ However, the effects of herb combinations on pig production were unknown. Thus, this study was designed to investigate the effects of three combinations of Chinese herbs on growth performance, nutrient digestibility, serum biochemical parameters, and immune function in swine.

### 4.1 Growth performance and nutrient digestibility

The results of this study demonstrate that three groups of Chinese herb feed additives enhanced the growth performance of pigs. The F/G of pigs in groups A, B, and C was decreased by 17.56%, 9.31%, and 13.86% compared with NC treatment. Furthermore, the results also demonstrated that the diets supplemented with Chinese herbs improved meat quality and the amino acid concentration of muscle, as the total protein, total amino acids, total lipid, and crude ash of pigs in group A, B, and C were increased.

Intestinal crypts are invaginations of the epithelium around the villi that are lined by epithelial cells that secrete enzymes. The base of each crypt is constantly dividing to maintain the structure of the villus.^12^, ^13^ Therefore, an increase in crypt depth produces more developed villi.^12^ This experiment showed that dietary supplementation with Chinese herb feed additive increased villus height:crypt depth in the ileum, jejunum, and duodenum compared to the NC (Figure 1), indicating that Chinese herbs can improve nutrient digestibility.

The results of this study indicate that the three groups of Chinese herb feed additives can improve the weight gain and meat quality of pigs, as well as the nutrient digestion and absorption capacity of the small intestine.

### 4.2 Serum biochemical parameters

The three groups of Chinese herb feed additives induced positive effects on serum biochemical parameters. The serum concentrations of TP, ALB, and BUN are commonly regarded as indicators of protein synthesis and metabolism, which are related to the growth performance in piglets, and BUN is the product of protein degradation in vivo.^8^, ^14^ In this study, serum TP and ALB levels were increased in the groups treated with Chinese herb feed additives compared with the control group, and serum BUN levels were decreased. Moreover, ALP is an important enzyme that contributes to the absorption of Ca and P and protein synthesis, and the serum GLU level is used as an indicator of energy supply ability.^8^, ^15^ Serum ALP and GLU levels were higher in the treatment groups than in the control group, indicating that the Chinese herbs, especially groups A and group B, can improve digestion and absorption in pigs. These results indicate that more proteins were synthesized and absorbed in the Chinese herb‐fed groups.

GTP is a transferase that catalyzes the transfer of gamma‐glutamyl functional groups from molecules such as glutathione to an acceptor that may be an amino acid, a peptide, or water.^16^ This transferase is found in many tissues and has been used as a diagnostic marker in liver.^17^ We found that serum GTP was decreased in the treated groups compared with the NC group. Furthermore, ALT and AST are enzymes related to liver function, and increased AST and ALT activity suggests liver disease, infection, parasitism, or trauma.^8^, ^18^ Serum ALT and AST levels in the A, B, and C groups were significantly decreased compared to the NC group, indicating that the Chinese herbs used in this study can protect the liver and have beneficial effects on liver synthetic function in swine.

TC, TG, HDLC, and LDLC are the indicators of serum cholesterol, which also correlates with the function and incidence of cardiovascular events. LDLC can build up on the walls of the arteries and increase the chance of heart disease, while higher HDL is correlated with cardiovascular health.^19^, ^20^ Serum TC and TG concentrations in the Chinese herb feed additive groups were significantly decreased compared to the NC groups (*P* < .05). Moreover, serum LDLC was lower than in the control group. There was no significant difference in HDLC between the Chinese herb‐treated groups and the control.

AMS is an enzyme that catalyzes the hydrolysis of starch into sugars. Higher blood serum amylase may reflect acute inflammation of the pancreas.^21^ In this study, serum AMS was lower than in the control group, indicating the Chinese herbs improved pancreas health. CREA is a product of creatine phosphate in muscle, and serum CREA is an important indicator of renal health.^22^ In this study, serum CREA was increased in the treated groups compared with the control group. Additionally, A/G was increased in the treated groups compared with the control group, while GLO was decreased.

Together, the results in this study suggested that diets supplemented with Chinese herb feed additives could induce positive effects on serum biochemical parameters and immune function in swine.

## 5 CONCLUSION

In conclusion, the diets supplemented with Chinese herbs improved meat quality, increased the amino acid concentration of muscle, increased the villus height:crypt depth ratio, and induced positive effects on serum biochemical parameters and immune function in swine. The combination of these effects contributed to better absorption of nutrients by the intestinal tract and yielded a better growth performance. This research will help guide the use of Chinese herbs as feed additive supplementation in swine diets.

## CONFLICT OF INTEREST

None.

## AUTHOR CONTRIBUTIONS

JYB and ZYY conceived and designed the study; ZNL, LY, ZWL, XSH, ZL, YQY, and HWX involved in acquisition, analysis, and interpretation of data. ZYY and JYB drafted the manuscript and involved in revising; ZNL, LY, ZWL, XSH, ZL, YQY, HWX, JYB, and ZYY gave the final approval. ZNL, LY, ZWL, XSH, ZL, YQY, HWX, JYB, and ZYY are accountable for all the aspects of the work and for ensuring that questions related to the accuracy or integrity of any part of the work are appropriately investigated and resolved. All the authors read and approved the final version of the manuscript.

## ETHICS APPROVAL

The animal protocol for this experiment was approved by the Animal Care Committee of Fujian Academy of Agricultural Sciences, China. Animals were maintained and processed in accordance with Fujian Academy of Agricultural Sciences Guide for the Care and Use of Laboratory Animals.

## References

[1] Li J . Current status and prospects for in‐feed antibiotics in the different stages of pork production – A review. Asian‐Australas J Anim Sci. 2017;30:1667‐1673. 28823126 10.5713/ajas.17.0418 PMC5666167

[2] Cheng DL , Ngo HH , Guo WS , et al. Bioprocessing for elimination antibiotics and hormones from swine wastewater. Sci Total Environ. 2018;621:1664‐1682. 29074241 10.1016/j.scitotenv.2017.10.059

[3] Kang SN , Chu GM , Song YM , Jin SK , Hwang IH , Kim IS . The effects of replacement of antibiotics with by‐products of oriental medicinal plants on growth performance and meat qualities in fattening pigs. Anim Sci J. 2012;83:245‐251. 22435629 10.1111/j.1740-0929.2011.00942.x

[4] Windisch W , Schedle K , Plitzner C , Kroismayr A . Use of phytogenic products as feed additives for swine and poultry. J Anim Sci. 2008;86(14 Suppl.):E140‐148. 18073277 10.2527/jas.2007-0459

[5] Murugesan GR , Syed B , Haldar S , Pender C . Phytogenic feed additives as an alternative to antibiotic growth promoters in broiler chickens. Front Vet Sci. 2015;2:21. 26664950 10.3389/fvets.2015.00021 PMC4672194

[6] Huang K‐C , Yen H‐R , Chiang J‐H , et al. Chinese herbal medicine as an adjunctive therapy ameliorated the incidence of chronic hepatitis in patients with breast cancer: a nationwide population‐based cohort study. Evid Based Complement Alternat Med. 2017;2017:1052976. 29234362 10.1155/2017/1052976 PMC5682887

[7] Haselmeyer A , Zentek J , Chizzola R . Effects of thyme as a feed additive in broiler chickens on thymol in gut contents, blood plasma, liver and muscle. J Sci Food Agric. 2015;95:504‐508. 24862829 10.1002/jsfa.6758

[8] Zhou H , Wang C , Ye J , Chen H , Tao R . Effects of dietary supplementation of fermented *Ginkgo * *biloba* L. residues on growth performance, nutrient digestibility, serum biochemical parameters and immune function in weaned piglets. Anim Sci J. 2015;86:790‐799. 25827443 10.1111/asj.12361

[9] Ao X , Meng QW , Kim IH . Effects of fermented red ginseng supplementation on growth performance, apparent nutrient digestibility, blood hematology and meat quality in finishing pigs. Asian‐Australas J Anim Sci. 2011;24:525‐531.

[10] Hossain ME , Ko SY , Park KW , Firman JD , Yang CJ . Evaluation of green tea by‐product and green tea plus probiotics on the growth performance, meat quality and immunity of growing‐finishing pigs. Anim Prod Sci. 2012;52:857‐866.

[11] Hossain ME , Yang CJ . Effect of fermented water plantain on growth performance, meat composition, oxidative stability, and fatty acid composition of broiler. Livest Sci. 2014;162:168‐177.

[12] Chwen LT , Foo HL , Thanh NT , Choe DW . Growth performance, plasma fatty acids, villous height and crypt depth of preweaning piglets fed with medium chain triacylglycerol. Asian‐Australas J Anim Sci. 2013;26:700‐704. 25049841 10.5713/ajas.2012.12561 PMC4093331

[13] Le MH , Galle S , Yang Y , et al. Effects of feeding fermented wheat with *Lactobacillus * *reuteri* on gut morphology, intestinal fermentation, nutrient digestibility, and growth performance in weaned pigs. J Anim Sci. 2016;94:4677‐4687. 27898943 10.2527/jas.2016-0693

[14] Yoon SY , Yang YX , Shinde PL , et al. Effects of mannanase and distillers dried grain with solubles on growth performance, nutrient digestibility, and carcass characteristics of grower‐finisher pigs. J Anim Sci. 2010;88:181‐191. 19749022 10.2527/jas.2008-1741

[15] Wang H , Chen YI , Zhao Y , et al. Effects of replacing soybean meal by detoxified *Jatropha* *curcas* kernel meal in the diet of growing pigs on their growth, serum biochemical parameters and visceral organs. Anim Feed Sci Tech. 2011;170:141‐146.

[16] Tate SS , Meister A . Gamma‐glutamyl transpeptidase from kidney. Methods Enzymol. 1985;113:400‐419. 2868390 10.1016/s0076-6879(85)13053-3

[17] Dominici S , Paolicchi A , Corti A , Maellaro E , Pompella A . Prooxidant reactions promoted by soluble and cell‐bound gamma‐glutamyltransferase activity. Methods Enzymol. 2005;401:484‐501. 16399404 10.1016/S0076-6879(05)01029-3

[18] Lander ME , Harvey JT , Gulland FM . Hematology and serum chemistry comparisons between free‐ranging and rehabilitated harbor seal (Phoca vitulina richardsi) pups. J Wildl Dis. 2003;39:600‐609. 14567222 10.7589/0090-3558-39.3.600

[19] Keene D , Price C , Shun‐Shin MJ , Francis DP . Effect on cardiovascular risk of high density lipoprotein targeted drug treatments niacin, fibrates, and CETP inhibitors: meta‐analysis of randomised controlled trials including 117,411 patients. BMJ. 2014;349:g4379. 25038074 10.1136/bmj.g4379 PMC4103514

[20] Glagov S , Weisenberg E , Zarins CK , Stankunavicius R , Kolettis GJ . Compensatory enlargement of human atherosclerotic coronary arteries. N Engl J Med. 1987;316:1371‐1375. 3574413 10.1056/NEJM198705283162204

[21] Smith RC , Southwell‐Keely J , Chesher D . Should serum pancreatic lipase replace serum amylase as a biomarker of acute pancreatitis? ANZ J Surg. 2005;75:399‐404. 15943725 10.1111/j.1445-2197.2005.03391.x

[22] Samra M , Abcar AC . False estimates of elevated creatinine. Perm J. 2012;16:51‐52. 10.7812/tpp/11-121 PMC3383162 22745616

